# Synthesis and Regulation of miRNA, Its Role in Oncogenesis, and Its Association with Colorectal Cancer Progression, Diagnosis, and Prognosis

**DOI:** 10.3390/diagnostics14131450

**Published:** 2024-07-07

**Authors:** Monika Rac

**Affiliations:** Department of Biochemistry and Medical Chemistry, Pomeranian Medical University, Al. Powstańców Wielkopolskich 72, 70-111 Szczecin, Poland; monika.rac@pum.edu.pl

**Keywords:** miRNA, gen regulation, oncogenesis, colorectal cancer

## Abstract

The dysfunction of several types of regulators, including miRNAs, has recently attracted scientific attention for their role in cancer-associated changes in gene expression. MiRNAs are small RNAs of ~22 nt in length that do not encode protein information but play an important role in post-transcriptional mRNA regulation. Studies have shown that miRNAs are involved in tumour progression, including cell proliferation, cell cycle, apoptosis, and tumour angiogenesis and invasion, and play a complex and important role in the regulation of tumourigenesis. The detection of selected miRNAs may help in the early detection of cancer cells, and monitoring changes in their expression profile may serve as a prognostic factor in the course of the disease or its treatment. MiRNAs may serve as diagnostic and prognostic biomarkers, as well as potential therapeutic targets for colorectal cancer. In recent years, there has been increasing evidence for an epigenetic interaction between DNA methylation and miRNA expression in tumours. This article provides an overview of selected miRNAs, which are more frequently expressed in colorectal cancer cells, suggesting an oncogenic nature.

## 1. Introduction

The study of the molecular mechanisms of cancer initiation and progression may provide a scientific basis for the development of new strategies for the prevention of tumour formation and the treatment of patients with cancer [1]. The dysfunction of several types of regulators, including miRNAs, has recently attracted scientific attention for their role in cancer-associated changes in gene expression [2]. MiRNAs are small RNAs of ~22 nt in length that do not encode protein information but play an important role in post-transcriptional mRNA regulation [3]. Studies have shown that miRNAs play a significant and complex role in tumourigenesis, influencing various aspects of tumour progression, such as cell proliferation, cell cycle, apoptosis, tumour angiogenesis, and invasion [4]. The biogenesis of miRNAs involves complex activities divided into canonical and non-canonical pathways. Many factors can affect the expression and function of miRNAs [5]. Dysregulation of miRNAs may be associated with tumourigenesis processes such as cell cycle dysregulation and immune evasion [6]. Because of their ability to modulate hundreds of specific genes, miRNAs are emerging as important biomarkers in cancer diagnosis or prognosis [7,8]. The detection of selected miRNAs may help in the early detection of cancer cells, and monitoring changes in their expression profile may serve as a prognostic factor in the course of the disease or its treatment [9]. Defining target genes for ‘dysregulated’ miRNAs in cancer cells and developing methods to selectively silence them is a promising therapeutic strategy for cancer patients [10,11].

## 2. MiRNA Synthesis

The formation of miRNAs is a complex process that starts in the nucleus and ends in the cytoplasm. Genes encoding miRNAs, located in exons or introns of protein-coding genes, also in intergenic regions of high instability, occur as single genes with their own promoter or as groups of genes with a common promoter [12]. MiRNA genes located within introns are transcribed by RNA polymerase, undergo splicing, and typically undergo two sequential cuts to generate primary miRNA (pri-miRNA) transcripts [13].

### 2.1. Canonical miRNA Biogenesis

**Figure 1:**
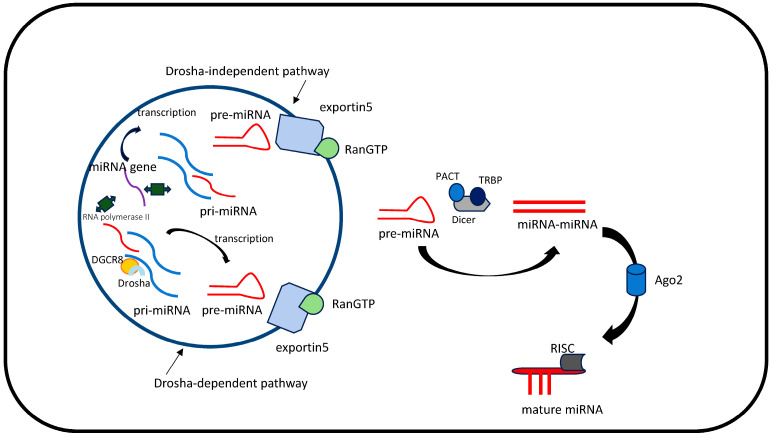
MiRNA biogenesis—canonical and non-canonical pathways.

In the canonical pathway, miRNA genes with their own promoter, like protein-coding genes, are transcribed by RNA polymerase II to form a primary transcript called a pri-miRNA (primary miRNA) [14]. This transcript contains a 7-methylguanosine cap at the 5′ end and a poly (A) tail at the 3′ end. In subsequent steps, the pri-miRNA molecule is recognised by a complex (known as the microprocessor) consisting of the ribonuclease Drosha (RNAase III) and DiGeorge syndrome critical region 8 (DGCR8, the RNA-binding protein) [15,16]. In the next step, the pri-miRNA is cleaved to release a fragment with a ‘hairpin’ structure called pre-miRNA (precursor miRNA), which is actively transported into the cytoplasm by exportin-5, which cooperates with RanGTP (Ran guanosine triphosphatase) [17]. The pre-miRNA/Exportin5/RanGTP complex releases pre-miRNA after the hydrolysis of RanGTP to RanGDP. The reaction is catalysed by the GTPase RanGAP [18]. The energy gained from GTP hydrolysis enables the transport of pre-miRNA into the cytoplasm. In the cytoplasm, the pre-miRNA is further recognised by Dicer, an endonuclease with two RNAase III domains, and binds to a protein called TRBP (HIV-1 TAR RNA-binding protein, also known as TARBP2) [19]. The pre-miRNA is then processed in the cytoplasm with the involvement of the endonuclease Dicer (RNAase III), which cuts the terminal loop from the hairpin and generates a miRNA–miRNA duplex, in which one miRNA strand is a delayed strand paired with the second miRNA [20]. The result is a duplex of complementary miRNA molecules about 22 nucleotides long. After cutting the loop, two miRNA-X molecules can be formed from the pre-miRNA precursor molecule, where X is the miRNA identification number. MiRNA-X-3p refers to the molecule formed on the 3′ arm, and miRNA-X-5p refers to the molecule formed on the 5′ arm of the pre-miRNA [21]. The Dicer enzyme is assisted by two proteins with similar structures, PACT (protein activator of protein kinase R) and TRBP (HIV-1 transactivating response RNA-binding protein), which stabilise it and facilitate the transfer of the mi-RNA duplex to the Argonaute (Ago) protein [22]. The miRNA–miRNA duplex then loads into an Ago protein, where the N-terminal domain of Ago unwinds it. The nucleotide composition of the miRNA–miRNA duplex influences strand selection during sorting among Ago complexes [23]. Typically, the lagging strand is degraded, while the leading strand matures into the functional miRNA and forms a functional RNA-induced silencing complex (RISC). Occasionally, a double-stranded miRNA is incorporated into an RISC, which recognises the leading strand as the mature miRNA (Figure 1) [24]. Therefore, mature miRNAs are generated in the cytoplasm. Although they are primarily in the cytoplasm, they can also exist in the cell nucleus.

### 2.2. Non-Canonical miRNA Biogenesis

There are alternative mechanisms of miRNA biogenesis. Unlike the canonical pathway, the non-canonical pathway does not require the Drosha complex and DGCR8 to produce pre-miRNA. Instead, the resulting structure is exported to the cytoplasm by exportin-5, where the Dicer complex recognises and cleaves it to form mature miRNA (Figure 1) [25]. Some miRNAs undergo non-canonical biogenesis, characterised by a Drosha-independent pathway. Drosha-independent miRNAs can be divided into three classes [26]. The first class includes ‘mirtrons’, which are specific introns that act as pre-miRNAs and are immediately exported from the nucleus for Dicer-mediated processing [27]. Mammalian mirtrons differ from Drosha-generated pre-miRNAs in several ways, including much longer mirtron hairpins, frequent 3′-uridylation, and unusually heterogeneous 5′ ends. The mirtron pathway is a constant class of non-canonical biogenesis. It allows miRNAs to appear prior to the appearance of Drosha [28]. The second class is derived from other ncRNAs, such as small nucleolar RNAs (snoRNAs) and transfer RNAs (tRNAs). The transcription of their genes depends on the activity of crucial transcription factors and the processing of various RNAs to help produce hairpins that serve as Dicer substrates [29]. However, although some tRNA and snoRNA fragments can form potential miRNAs and even be loaded into RISCs, most of the tRNA and snoRNA fragments associated with Ago may remain unidentified [30]. For example, studies suggest that DNA damage response DDR) may be mediated by such ncRNAs [31]. In addition, tRNA-derived fragments have been shown to increase mRNA translation by regulating ribosomal proteins such as RPS28 [32]. The third class includes 7-methylguanosine (m7G) pre-miRNAs, which are synthesised directly from the 5′ ends of their genes by Pol II. The next step is their export to the cytoplasm by exportin 1 [33]. Dicer-independent miRNAs are much less numerous compared to Drosha-independent ones. This method of miRNA biogenesis requires the catalytic activity of the Ago2 protein [34].

## 3. Mechanism of Regulation by miRNA

**Figure 2:**
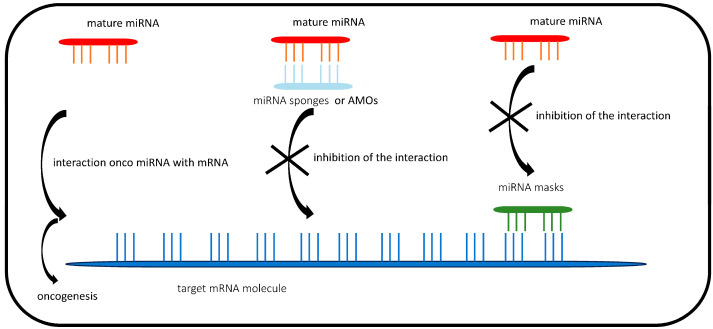
Inhibition of target mRNA by onco-miRNAs and the oncogenic miRNA inhibition strategy.

MiRNAs regulate gene expression by binding to mRNAs. Mature miRNA molecules incorporated into an effector RISC complex (miRISC, RISC with incorporated miRNA, or miRNA-induced silencing complex) are able to bind to the target mRNA. In this way, they induce mRNA degradation and translation silencing (Figure 2) [24]. A seed sequence is required for miRNA to bind to mRNA. In a mature miRNA molecule, there is a conserved 7-nucleotide heptameric sequence at the 5′ end. It is most commonly located between nucleotides 2 and 7, counting from the 5′ end of the miRNA. This fragment is known as the “seed” region. The association of the ‘seed’ region of the miRNA molecule with miRNA response elements (MREs) located at the 3′(UTR) end of the target mRNA leads to endonucleolytic degradation of the transcript by Ago2. This protein is a member of the Argonaute family of proteins. Although the base pairing of the miRNA and its target mRNA is not perfect, the ‘seed sequence’ must be perfectly complementary [35]. The mRNA can be degraded from the 3′ to the 5′ end (due to the activity of the exosome catalytic complex) or from the 5′ to the 3′ end (catalysed by the exonuclease Xrn1p). The effect is to down-regulate a specific degradable transcript or to inhibit the synthesis of the protein it encodes. The inhibition of translation can occur at the initiation or elongation stage [36]. This is because the binding of miRISC to the target transcript prevents the binding of the transcription initiation factor (eIF4e) to the 5′ cap, thereby preventing the initiation of translation. Another mechanism of translation repression by miRNAs is to prevent the attachment of ribosome subunits to the target mRNA. This involves the attachment of the 60S subunit to the 40S subunit in the presence of the transcript. The binding of the miRISC complex also prevents mRNA circularisation, resulting in reduced translation efficiency. MiRNAs indirectly affect premature ribosome dissociation, which can lead to transcript accumulation in the P bodies [37]. MiRNAs target the 3′UTR site of mRNAs encoding transcriptional regulators, such as methylation and transcription factors, to induce changes in expression [38]. If a specific miRNA changes the expression of an mRNA, this can, in turn, change the expression of other miRNAs. The formation of miRNA–mRNA–miRNA networks can be used to identify the master miRNA regulator that controls the expression of most miRNAs in the network [39]. It also allows the identification of the most important regulatory miRNAs and the arrangement of miRNAs in their hierarchy of action. It is also possible to edit mRNAs as a post-transcriptional mechanism by which miRNAs modify specific nucleotides at the mRNA level. In addition, miRNAs can bind to other miRNAs and non-coding RNAs. This leads to transcriptional silencing, including the silencing of other miRNAs. In the same way, miRNA modulates mRNA expression by controlling its transcriptional or regulatory pathways within the gene regulatory network. Thus, the miRNA–miRNA interaction is due to secondary transcriptional control rather than a direct interaction [40]. Interestingly, miRNAs can regulate their own expression by controlling the immature form. Therefore, miRNA–miRNA interactions may also play a role in autoregulation. Single nucleotide polymorphisms (SNPs) in the seed region of miRNAs are another factor that has an impact on changes in miRNA expression [41]. The seed sequence is essential for binding to the target mRNA or the target recognition sequence [42]. Changes in either of these domains can lead to a loss of target regulation. In addition, different miRNA isoforms can alter the seed region by adding nucleotides from the 5′ or 3′ end of the miRNA. In this way, they affect the targeting of selected genes and play a role in disease progression. The presence of SNPs or miRNA isoforms that alter the seed region can potentially alter both miRNA and mRNA expression, with cascading effects on the cellular environment [43].

On a genome-wide basis, the loss or gain of super-enhancers has extensive implications for miRNA and gene expression. Super-enhancers are genomic loci containing multiple enhancer elements that cooperatively bind multiple transcription factors. Under normal physiological conditions, super-enhancers control gene transcription and miRNA transcription that is specific to a particular cell type [44]. If they are altered, this leads to a loss of cell specificity, typical for carcinogenesis. A decrease in the level of miRNAs that are specific to a particular cell type leads to an increase in the level of other miRNAs that were previously expressed at lower levels. Consequently, this altered miRNA level itself controls a different set of genes. This further increases the potential for oncogenic changes [45]. Typically, the loss of super-enhancer regions leads to an increase in tumour suppressor miRNAs, while the gain of super-enhancers enriches oncogenic miRNAs. In exceptional cases, miRNA binding to mRNA can lead to the activation of the translation of the target transcript.

## 4. How Do miRNAs Play a Role in Oncogenesis?

There is currently no single mechanism or theory for miRNA dysregulation in cancer. Given the complexity of the regulation of human metabolism, it is possible that multiple mechanisms are involved, including those involving miRNA expression, the regulation of transcription factors, and mutations within miRNA strands. In addition, mutations in the miRNA transporter gene from the nucleus to the cytoplasm can lead to a loss of exportin-5 function, which means that pre-miRNAs are not transported to the cytoplasm, resulting in reduced levels of mature miRNAs in the cytoplasm [46]. One mRNA molecule can be inhibited by several miRNAs. The effect of the interaction with target mRNA molecules depends on the complementarity of the binding and the expression level of the miRNA or mRNA. Abnormalities in miRNA expression, either qualitative (e.g., lack of expression of a particular miRNA or expression of a miRNA not previously present in that tissue) or quantitative (increase or decrease in expression of selected miRNAs), have been shown to occur in many diseases, including cancer. Dysregulation of miRNA expression is closely associated with cancer initiation, progression, and metastasis. By analysing miRNAs from healthy and cancerous samples from humans, the researchers found that miRNA expression is globally downregulated in cancer cells compared to normal cells. In addition, the expression of miRNAs themselves can be altered in the tumour itself, during tumour progression, and in different types of cancer [47,48].

MiRNAs regulate the expression of approximately 60% of human genes. Since miRNAs are required to maintain the normal regulation of cellular processes, such as protein synthesis, metabolism, and cell proliferation, under normal physiological conditions, their deregulation leads to abnormal cell growth and metabolism, which contribute to cancer development, progression, and metastasis. The miRNA-mediated control of gene expression is critical in the cellular response to environmental stresses such as oxidative stress, hypoxia, starvation, and DNA damage [49]. Depending on their function in cancer development, miRNAs are classified as oncogenes, activating oncogenesis or inhibiting the expression of suppressor genes (‘oncomiRNAs’), or tumour suppressors, inhibiting the expression of oncogenes or apoptosis-inducing genes (‘oncosuppressor miRNAs’) [50]. Some miRNAs (miRNA-21, miRNA-17∼92 cluster, and miRNA-200 family) are packaged into exosomes, secreted from the cell, and can act in different ways depending on the target tissue [51,52]. Therefore, modulation of the tumour microenvironment is responsible for the heterogeneity of patient response to treatment. Tumour angiogenesis can be controlled by miRNAs such as miRNA-210 and miRNA-519c under hypoxic conditions by modulating the vascular endothelial growth factor (VEGF) and hypoxia-inducible factor 1α (HIF-1α) [53]. Reduced or absent expression of tumour suppressor miRNAs results in the increased expression of genes important for tumour progression, including anti-apoptotic proteins or transcription factors. For many miRNAs (e.g., miRNA-155 and miRNA-125b), the effect of their activity depends on the cumulative activity of the regulated genes [54]. The expression of tumour suppressor miRNAs depends on DNA methylation but can also affect the activities of epigenetic regulators such as DNA methyltransferases or histone deacetylases [55].

Changes in miRNA expression in tumours can result from alterations in genomic miRNA loci. Expression of miRNAs is also controlled at the transcriptional level, mediated by the epigenetic control of DNA methylation and transcription factors [56]. For example, the expression of miRNA-34 family genes (miRNA-34a, miRNA-34b, and miRNA-34c) is controlled by the transcription factor p53, reflecting the importance of the functional status of p53 in predicting miRNA-34 expression in human cancers [57]. Among other functions, the p53 protein is involved in the PI3K–AKT signalling pathway. Therefore, the disruption of this pathway leads to p53 instability and dysfunction, which affects cancer development [58]. Following DNA damage and oncogenic stress, p53 is activated and regulates miRNA-34 transcription, which affects cell cycle arrest, apoptosis, and ageing [59]. In contrast, the miRNA-143/145 cluster is repressed by oncogenic RAS signalling, which induces tumourigenesis. The RAS-responsive element binding protein 1 (RREB1) leads to the transcriptional repression of the miRNA-143/145 cluster, and miRNA-143/145, in turn, represses RREB1 expression, creating a cancer-promoting feedback loop of RAS signalling [60]. In turn, miRNA–miRNA interactions are integrated into pathways that are critical for cancer progression. These are interactions between miRNA-205 and miRNA-184, which affect levels of the SH2 lipid phosphatase containing phosphoinositide 5′-phosphate 2. Both miRNAs have overlapping binding sites within the 3′UTR of SHIP2, with miRNA-184 mediating SHIP2 repression by miRNA-205 blocking access to the binding site without inducing upregulation [61]. This has implications for cell growth, proliferation, and apoptosis due to the involvement of SHIP2 in the AKT pathway, suggesting that this miRNA–miRNA interaction is a major contributor to the tumour phenotype [62]. Several miRNAs may act in a cooperative manner. The concept of miRNA synergism assumes the existence of a “master regulator” of miRNAs so that any changes in the expression of a master regulator miRNA would also alter the miRNA in its synergistic network. Similarly, miRNAs that target transcriptional regulators may alter the transcriptional activity of miRNAs with similar functions to contribute to a coordinated response. Hormones (estrogen, progesterone, androgens, glucocorticoids, and mineralocorticoids) can also regulate miRNA expression in tumours by binding to nuclear receptors [63]. Therefore, targeting or activating specific transcription factors responsible for the diversity of onco-miRNAs or onco-suppressor miRNAs may be a promising and innovative approach to cancer treatment.

## 5. Using miRNAs for Diagnosis and Treatment

MiRNAs have been identified as potential markers of cancer. Firstly, miRNA molecules are readily available for study as they are present in all body fluids. Secondly, the high biological stability of miRNAs makes them easy to detect. Thirdly, miRNAs regulate all stages of tumour progression and, in many cases, show tissue-specific expression. The use of miRNAs as predictive and prognostic biomarkers during treatment appears to be particularly clinically relevant. The expression of miRNAs in chemoresistant tumour cells may differ from that in chemosensitive cells [64,65].

Going forward, it would be important to develop one of two therapeutic strategies using miRNAs that could potentially inhibit tumour growth. One is to inhibit oncogenic miRNAs. The other is to re-establish the expression of tumour-suppressing miRNAs, which are silenced in cancer cells. The strategy of importing exogenous miRNAs may play a role in cancer treatment by inhibiting proliferation or inducing apoptosis of tumour cells [66]. The inhibition of oncogenic miRNAs involves the introduction of miRNA-mimicking molecules into tumour cells. Several variants of inhibitory molecules have been tested, including miRNA sponges, miRNA masks, antagomirs, anti-miRNA oligonucleotides (AMOs), antisense oligonucleotides using ‘locked’ nucleic acids (AMOs), anti-miRNA oligonucleotides (AMOs), and anti-miRNA oligonucleotides (AMOs). The principle of their action is to inhibit the interaction of miRNAs with each other and with other antisense oligodeoxyribonucleotides (MTg-AMOs) [67,68,69,70]. Their principle of action is to inhibit the interaction of the oncogenic miRNA with the target mRNA. MiRNA inhibitors are transcripts containing sites that mimic sequences found in mRNAs complementary to the target miRNA. The use of miRNA inhibitors reduces the number of free miRNAs (of one or more types) by binding them to the inhibitor molecule instead of the target mRNA. This leads to an increase in the expression of the primarily inhibited mRNAs (Figure 2).

The limitation to the potential use of miRNA molecules in therapy is the lack of efficient methods to deliver synthetic miRNAs into the tumour environment. Chemically synthesised miRNAs could be introduced into tumour cells using different types of transporters, e.g., liposomes [71]. However, to develop an effective method of delivering synthetic miRNA variants, they need to be protected from early degradation in the bloodstream, then delivered directly to the target cells, and prevented from triggering an immune response. A safer solution is to deliver miRNA molecules directly to the tumour, which can lead to the silencing of target genes with reduced toxicity. This method is more effective than the systemic delivery of synthetic miRNAs but is limited to easily accessible lytic tumours [72]. It should also be recognised that testing one selected miRNA molecule is insufficient in most cases, as changes in tumour cells depend on the expression of different miRNAs with pleiotropic effects [73].

## 6. Signalling Pathways and Possible Therapeutic Strategies in Colorectal Cancer

At present, the treatment of CRC mainly includes surgery, radiotherapy, chemotherapy, immunotherapy, and targeted therapy. However, approximately 50% of CRCs are resistant to 5-Fu-based chemotherapy regimens, and immunotherapy is effective only for 10–15% of patients with CRC [74]. Therefore, it is necessary to find new and effective therapeutic strategies for improving drug resistance in CRC. Certain miRNA-mediated signalling pathways play a crucial role in the progression and metastasis of CRC cells. This involvement also explains why miRNAs contribute to drug resistance in patients with CRC. An effective treatment regimen requires the selection of the right regulatory molecules and the selection of appropriate signalling pathways. Moreover, the nanoparticle carriers have opportunities for efficiently delivering miRNA therapeutics with less toxicity compared with other anticancer drugs [75,76].

Gastrointestinal epithelial cells are known for constant proliferation and differentiation, making changes in signalling pathways more likely to increase the likelihood of carcinogenesis [77]. The release of inflammatory mediators, including chemokines, cytokines, and stromal or epithelial cells, contributes to the activation of carcinogenesis-related pathways such as Wnt/β-catenin, Notch, JAK/STAT, PI3K, NFkB, TGF-β, and Hedgehog. Recurrent inflammatory bowel disease, together with epigenetic changes, may ultimately lead to the development and progression of colorectal cancer (CRC). CRC develops through the following pathway: inflammation–cell dysplasia–cancer [78]. However, we do not yet fully understand the epigenetic changes in cellular metabolism that occur during tumour progression.

Dysregulation of the Wnt signalling system leads to colonic epithelial dysfunction [79,80]. As a regulatory pathway regulating colorectal development, hyperactivation of the Wnt/β catenin pathway is found in more than 90% of CRC cells [81]. When Wnt stabilises β-catenin and binds to the LRP target complex, its downstream targets are phosphorylated. The activation of the Wnt cascade is responsible for cell polarity, proliferation, and differentiation. Wnt/β-catenin signalling cross-talks with MAPK, Notch, and TGF-β signalling [82].

The NOTCH signalling pathway, which causes ALDH1A1 protein deacetylation, is crucial for goblet cell control and progenitor cell differentiation. Notch signalling cascade components are expressed in intestine bottom crypts and colonic mucosa cells. The overexpression of Notch pathways in cancer is linked to poor prognosis and aggressive metastasis [83].

The cytokine receptor binding and phosphorylation of the JAK/STAT pathway lead to related gene regulation. Genetic mutations in the JAK/STAT pathway lead to aberrant activation, even without cytokine induction, which results in persistent activation and tumourigenesis. Overexpression is revealed by the circular RNA circSPARC, some lncRNAs, and miRNAs [84].

The phosphatidylinositol-3-kinase (PI3K) signalling pathway is a group of related enzymes that help cells grow, divide, differentiate, move, stay alive, and move around inside other cells. PI3Ks send signals inside cells as phosphorylation of the hydroxyl group at position 3 of the inositol ring of phosphatidylinositol. Activation of PI3K occurs by binding inflammatory mediators (e.g., TNF-α, IL-1β, IL6, and STAT3) to their respective receptors in cells present in the colon (Paneth cells, macrophages, enterocytes, and cup cells. This leads to the activation of AKT-mTOR and SGK. In this way, PI3K regulates the cell cycle by controlling the growth, proliferation, and apoptosis of colon cells. This signalling pathway influences the sensitivity of cancerous tumours to insulin and IGF1 [85]. Mutation analysis in patients with CRC indicates a sense variant in the 3`UTR region of the IL23R gene (rs10889677) and induces PI3K [86]. Hence, the control of PI3K/AKT pathway regulation may be a therapeutic target in CRC.

NF-κB (nuclear factor-kappa B) is a transcription factor that mediates a cytoplasmic/nuclear signalling pathway and regulates the gene expression of various cytokines, cytokine receptors, and adhesion molecules involved in inflammatory and immune reactions. Upon activation, the NFκB signalling pathway complex is phosphorylated and induces the phosphorylation of other proteins, leading to NFκB freely translocating into the nucleus to regulate the expression of their target genes. NFκB is the transcription factor family of five subunits: Rel (cRel), RelB, p105/p50 (NFκB1), p100/p52 (NFκB2), and p65 (RelA, NFκB3). These heterologous or homologous dimers can bind to a specific DNA sequence, named NFκB sites, of the target gene to regulate gene transcription. During inflammatory events via NF-κB activation, the levels of inflammatory cytokines increase. Over-activation of the NF-κB pathway is a feature of colorectal cancer (CRC). When NF-κB binds to the cytoplasmic inhibitor I-kappa B (IκB), NF-κB is inactivated, preventing its DNA binding and nuclear uptake. Therefore, combining chemotherapeutic agents with NF-κB inhibitors can increase chemosensitivity in CRC cells [87,88].

The TGF-β signalling pathway is crucial for cell cycle inhibition and tissue homeostasis. When TGF-β ligands attach to their receptors, the receptor–ligand heteromeric complex assembles and autophosphorylates. Inhibitory SMADs and SMAD-ubiquitinated regulatory factors can destroy TGF–receptor complexes. It leads to damaged cells in the digestive tract and is crucial for tumour invasion and metastasis development. The TGF-β signalling pathway hyperactivation encourages angiogenesis and immunosuppression. A role in the immune response is played by the Eomesodermin/Tbr2 protein (EOMES), which has a DNA-binding domain in the T-box gene region and encodes transcription factors that control gene expression and are involved in the regulation of the cell cycle. The tbr2 gene is vulnerable to microsatellite instability, frameshift mutations, and replication errors. That is why the TGF-β signalling pathway is one of the most frequently changed cell signalling cascades observed in many cancer types, and accounting for 40% to 80% of all colon cancer cases [89].

The dysregulation and hyperactivation of the hedgehog (Hh) signalling system can increase inflammation processes and lead to tumour growth in various tissues, including CRCs. For Hh signalling to happen, the Gli gene needs to be turned on. This gene belongs to the family of zinc finger transcription factors, which has three homologues: Desert Hedgehog (Dhh), Indian Hedgehog (Ihh), and Sonic Hedgehog (Shh). RASP acylation of the H-N-terminus is what starts the cascade. The Hh–Gli pathway is overactive in CRC stem cells, as in primary human colon carcinoma cells [90].

The MAPK/ERK pathway transmits a signal from a cell surface receptor to the DNA in the cell nucleus. There are many proteins in the pathway, including mitogen-activated protein kinases (MAPKs) or extracellular signal-regulated kinases (ERKs). The proteins talk to each other by phosphorylating nearby proteins, which turns them into active or de-active ones. Abnormalities in the MAPK signalling pathway are crucial in the carcinogenesis process, as are cell differentiation, proliferation, invasion, angiogenesis, apoptosis, and metastasis [91].

Alterations in miRNA expression, including the disruption of key signalling pathways and over-expression, may play a critical role in angiogenesis, metastasis, and drug resistance in CRCs. Available therapeutic strategies for the treatment of inflammation-associated CRC include the use of monoclonal antibodies that block pro-inflammatory molecules. Nucleic acid-based drugs (siRNAs or miRNAs) can be used to suppress oncogenic gene expression or activate tumour suppressors. In addition, competitive inhibitors of molecules in the inflammatory signalling cascade, such as tyrosine kinase inhibitors, may be used [92]. Because miRNAs play crucial roles in regulating signalling pathways, treatment may modulate miRNAs implicated in signalling pathways and carcinogenesis. MiRNAs are involved in resistance to therapeutic strategies in cancer [93]. Table 1 summarises the signalling pathways and their corresponding miRNAs.

To improve the efficacy of adjuvant therapy in colon cancer, six miRNAs (miRNA-21, miRNA-20a, miRNA-103a-3p, miRNA-106b, miRNA-143, and miRNA-215) are under clinical investigation (ClinicalTrials.gov identifier: NCT02466113). The function of the synthetic miR-193a-3p mimic 1B3 was tested (a phase 1 clinical trial) in cell lines derived from, among others, colon cancer. The use of 1B3 resulted in the downregulation of many oncogenic pathways in these cancer-derived cells, leading to suppressing cell proliferation and inducing apoptosis. INT-1B3 is currently being tested to determine the pharmacokinetics, safety of a maximally tolerated dose, pharmacodynamic response, and antitumour activity in patients with various solid cancers [113]. MiRNA-149 and miRNA-320 affect the Wnt/β-catenin signalling pathway and glucose metabolism, thereby increasing the sensitivity of 5-fluorouracil. In contrast, miRNA-135b, miRNA-182, and miRNA-587 can promote 5-FU resistance in CRC by activating the PI3K–AKT pathway. MiRNA-103 and miRNA-107 promote the Wnt/β-catenin signalling pathway to induce oxaliplatin resistance in CRC, but miRNA-506 overexpression inhibits this signalling pathway and enhances sensitivity to oxaliplatin. MiRNA-128-3p suppresses EMT and increases intracellular oxaliplatin accumulation, but miRNA-46146 induces oxaliplatin chemoresistance. The overexpression of miRNA-146a, miRNA-194, miRNA-451, miRNA-514b-3p, and miRNA-519c can increase the sensitivity of CRC to irinotecan. The upregulation of miRNA-100/125b promoted Wnt/β-catenin signalling and mediated resistance to cetuximab in CRC [93,114,115]. Since miRNAs are also responsible for regulating the expression of glycolysis enzymes, one therapeutic idea is based on the possibility of regulating these miRNAs in CRC cells [116,117,118,119]. In the case of research on cell lines, miRNA-140-3p, miRNA-382-3p, miRNA-148a-3p, miRNA-93-5p, miRNA-200a-3p, miRNA-200c-3p, miRNA-138-5p, and miRNA-15b-5p reduced tumour microsatellite instability. MiRNAs can target this immune checkpoint of MSI-H CRC and improve the efficiency of tumour immunotherapy [120]. MiRNAs show potential in the diagnosis, prognosis, and treatment of CRC, but their application in clinical practice requires more extensive research.

## 7. MiRNAs Role in Colorectal Cancer

Analytical methods based on miRNA identification are very important for diagnostic goals. Relative miRNA levels, and consequently mRNA levels, play an important role in carcinogenesis [121]. A better understanding of the underlying modulation of miRNA expression may, in the long term, allow the development of new molecular therapeutic interventions for patients with CRC [122]. To use miRNAs as biomarkers for cancer diagnosis and prognosis, it is important to correlate miRNA levels in the tumour cell, healthy tissue, and plasma [123]. The therapeutic targeting of non-coding RNAs is a new option for clinicians that is currently being evaluated [124].

The human intestinal epithelium is a rapidly renewing tissue in the body, and its homeostasis is tightly controlled by numerous factors at multiple levels [125,126]. Mi-RNAs may serve as diagnostic and prognostic biomarkers, as well as potential therapeutic targets for CRC. In recent years, there has been increasing evidence for an epigenetic interaction between DNA methylation modification and miRNA expression in tumours. The increased expression of selected miRNAs is more frequently observed in colorectal cancer cells, suggesting that they are oncogenic in nature. Increased miRNA expression may result from the amplification of genes encoding miRNAs but also from more efficient biogenesis, constitutive activity of their promoters, or increased stability of miRNA molecules [127]. Increased miRNA expression leads to the repression of many suppressor genes. Pri-miRNA transcription is under epigenetic control at the level of promoter-associated CpG island methylation [128]. Consequently, CRC cells observe hypermethylation of the miRNA-124-1 promoter region, leading to epigenetic repression of miRNA-124-1 loci and subsequent activation of its target [129]. The oncogenic miRNA miRNA-21 has been implicated in several miRNA–miRNA interactions. In colon cancer, tumour promotion occurs through indirect regulation of miRNA-145 expression [130]. An increase in miRNA-21 initiates the K-Ras signalling pathway. This activates the transcription factor RREBP (RAS responsive element binding protein) [131]. The RREB1 protein, in turn, inhibits miRNA-145 transcription. Therefore, the increase in miRNA-21 levels observed in tumours results in reduced miRNA-145 expression, which promotes oncogenic changes. MiRNA-195 is a key regulator of intestinal epithelial homeostasis in health and disease [132]. It targets several mRNAs encoding proteins involved in proliferation and migration. Increased levels of miRNA-195 also inhibit IGF signalling by reducing IGF2 receptor translation [133]. The overexpression of miRNA-195 inhibits the rapid recovery of the intestinal epithelium after acute injury. This is achieved by silencing the mRNA encoding Stim1, a protein required for the release of cell-stored Ca^2+^ into the cytoplasm [134]. Both human antigen R (HuR) and miRNA-195 target the 3′-UTR of Stim1 mRNA and antagonistically alter the deacylation of Stim1 mRNA [135]. As a result, they control cellular Stim1 levels in response to stress. The interaction between miRNA-195 and HuR also regulates the function of tuft cells (chemosensory cells in the epithelium) by altering the expression of cortactin-like kinase 1 (DCLK1) [136]. The deregulation of Tuft cells by miRNA-195 and the inactivation of HuR contribute to the disruption of the epithelial defence system [137,138]. Altered HuR expression and the deregulation of its affinity to bind mRNA are common in gastrointestinal cancers, particularly colorectal cancer [139]. However, the exact role of HuR in carcinogenesis is cell type dependent. HuR increases angiogenesis and induces metastasis in CRC cells. In addition, HuR upregulates the expression levels of cyclin D1, c-Myc, Bcl-2, and COX-2 (cyclooxygenase 2) and increases the activity of the Wnt/β-catenin, NF-κB, MAPK, and mTOR signalling pathways. All these factors and pathways are involved in the pathogenesis of cancer. HuR is also involved in chemoreception in CRC. It inhibits transport cytotoxic factors and drugs into cells. This inhibits apoptosis. [140,141]. Induced HuR expression is associated with weak overall survival rates in patients with gastric or colorectal cancer. MiRNA-29a/b is highly expressed in the intestinal epithelium. Its tissue levels change significantly with intestinal epithelial cell atrophy and intestinal barrier dysfunction [142]. A transcription factor named Jun increases HuR expression by inhibiting transcription of the miRNA-22 gene through specific AP1 binding sites in its promoter region. Additionally, miRNA-16 regulates HuR in the intestinal epithelium. Increased levels of miR-16 inhibit HuR expression at the translational step, resulting in the inhibition of COX-2 expression in colon tumours. The hypermethylation of some miRNA promoters (let-7, miRNA-9, miRNA-34, miRNA-129, miRNA-137, miRNA-342, and miRNA-345) also leads to a reduction in their expression and the development of CRC [143,144]. The aggressive CRC phenotype is significantly associated with miRNA-29b gene depression. The presence of miRNA-29b reduces the growth and invasion of tumour cells, with possible interaction with interferon-γ (IFN-γ), IRF1, and IGF1 [145]. On the other hand, the overexpression of miRNA-29b leads to growth inhibition of CRC cells in the G1 phase [146]. MiRNA-29a and miRNA-29b interact with mRNAs encoding cyclin-dependent kinase 2, PTEN, HMGB1, ZO-1, and claudin-1 through their 3′-UTR and reduce the stability and translation of their mRNAs. The interactions of miRNA-29 with these transcripts are negatively regulated by HuR. MiRNA-519 is downregulated in CRC, while its overexpression inhibits the migration, proliferation, and invasion of these tumour cells by suppressing Wnt/β-catenin signalling activity. Interestingly, miRNA-519 binds directly to HuR mRNA, resulting in the repression of HuR translation. This downregulation of HuR by miRNA-519 is associated with a decrease in the expression levels of HuR target mRNAs and the inhibition of cell proliferation [147]. Furthermore, miRNA-22 levels are also reduced in colorectal cancer. This is important because while miRNA-22 is induced, HuR translation is inhibited through an interaction with the 3′ UTR of HuR mRNA. This contributes to the inhibition of tumour growth [148].

As we know, RALY is an RNA-binding protein that is critical for regulating the Drosha complex and regulating the expression of specific miRNAs: miRNA-483, miRNA-676, and miRNA-877. By controlling miRNA expression, RALY regulates the mitochondrial signalling of proliferation or apoptosis in a cell under stress associated with ROS production. In patients with CRC, high levels of RALY protein are associated with a poor prognosis. Therefore, inhibiting RALY-specific adenosine methylation in a miRNA target loop recognition domain may prevent CRC progression in vivo [149]. MiRNA-124 suppresses colorectal cancer proliferation, migration, and invasion, but promoter methylation significantly downregulates its expression in colorectal cancer tissues [150]. Additionally, circulating miRNA-21 levels could be valuable for early detection and might serve as a novel diagnostic biomarker for CRC [151]. In turn, Homo sapiens circular RNA_0003602 (hsa_circ_0003602) directly interacts with miRNA-149-5p to negatively regulate its expression. MiRNA-149-5p binds directly to the 3′ untranslated region of SLC38A1, inducing its degradation and thereby reducing the malignant potential of CRC cells. Consequently, silencing circ_0003602 suppresses CRC cell viability, migration, invasion, angiogenesis, and glutaminolysis, induces cell apoptosis in vitro, and inhibits tumour growth in vivo [152].

Recently, miRNAs in the host have been regulated by the gut microbiota to influence host health [153]. For example, *Fusobacterium nucleatum* can exacerbate chemoresistance in CRC by reducing the expression of miRNA-18a and miRNA-4802 [154]. Furthermore, miRNAs structure the composition of the gut microbiota, ultimately influencing host physiology and disease [155,156]. MiRNA-515-5p and miRNA-1226-5p can promote the growth of *Fusobacterium nucleatum* and *Escherichia coli*, which have been reported to promote colorectal cancer progression [157,158,159,160,161].

Altered miRNA expression in the circulation may be a potential diagnostic or prognostic biomarker [162]. For example, serum levels of miRNA-823 are positively correlated with the CRC stage, with significantly higher levels observed in advanced stages (III and IV). Decreased expression of miRNA-28876 and miRNA-5937 correlates with early stage (TNM stages I-II), suggesting a role for these miRNAs in CRC staging. Increased expression of miRNA-21 (circulating in the blood) is strongly associated with a predisposition to colorectal cancer [163]. In colorectal cancer, increased miRNA-21 expression correlates with resistance to fluorouracil therapy through decreased expression of the repair protein MSH2. In vitro studies have shown that increased expression of miRNA-20a, miRNA-140, miRNA-215, and miRNA-224 promotes the development of chemoresistance to fluorouracil, oxaliplatin, methotrexate, or teniposide in CRC cells [164,165,166,167]. The miRNAs miRNA-5937, miRNA-28876, miRNA-23210, and miRNA-32159 were significantly downregulated in serum samples, with miRNA-5937 and miRNA-28876 specifically present only in patients with CRC [168]. The suppression of miRNA-823 inhibits cell proliferation, induces apoptosis, and arrests the cell cycle in the G1 phase in CRC cell lines. Furthermore, miRNA-823 expression was significantly upregulated in CRC tissues compared to adjacent tissues. Overexpression of miRNA-1245 and miRNA-24000 correlated with weak differentiation, the presence of distant metastases, and a higher stage of CRC [169]. Expression profiling of some miRNAs [45], miRNA-21, miRNA-31, miRNA-125b, miRNA-135b, miRNA-181a-2, miRNA-214, miRNA-222, and miRNA-335, allows stratification of benign colonic mucosal lesions according to their pathogenic potential. Using high-throughput sequencing (HTS), five miRNAs were identified with differential serum expression in patients with CRC compared to healthy controls: miRNA-001311, miRNA-004153, miRNA-017723, miRNA-017724, and miRNA-020365. It was observed that decreased levels of miRNA-017724 were associated with overall or progression-free survival from CRC. There was a significant increase in the expression of miRNA-54265 in serum samples from patients with CRC, which specifically binds to the PIWIL2 (Piwi Like RNA-Mediated Gene Silencing 2) protein, forming a PIWIL2/STAT3/phosphorylated SRC complex that promotes proliferation and metastasis by phosphorylating STAT3 [170]. Therefore, miRNA-54265 may serve as an important biomarker of disease modulation and dynamics. The presence of miRNA-124-3 (as an indicator of DNA methylation in the LOC386758 and SFRP1 genes) in laparoscopic colon fluid has a specificity of 79% and a sensitivity of 82% as a biomarker for the detection of colorectal cancer [171]. However, only one predictive diagnostic test, miRNApredX-31-3p, has been produced (IVD-certified) to date. It can be used in patients with CRC without mutations in the K-RAS gene [172]. Evaluation of miRNA-31-3p expression is performed in histopathological sections of colorectal tumours. Low miRNA-31-3p expression predicts higher clinical efficacy with anti-EGFR therapy compared to conventional chemotherapy. Replacement therapy is also being tested in CRC. This is an attempt to restore miRNA-34a expression, which is expected to lead to the inhibition of tumour progression and induction of programmed cell death [173]. In the case of CRC, the injection of synthetic miRNA let-7 may also contribute to the increased apoptosis of cancer cells. It has been observed that the local application of synthetic miRNA-145 induces an anti-tumour effect in mouse models of colon cancer [32]. Table 2 shows the list of miRNAs in patient serum and tumour tissue associated with the diagnosis of colorectal cancer progression.

## 8. Looking to the Future

In the future, it will be important to develop tests that provide valuable therapeutic targets, not just potential prognostic indicators. It is known that intravenous injection of lipid nanoparticles containing miRNAs can significantly reduce tumour progression [176,177]. A combinatorial strategy of anti-cancer miRNAs with chemotherapeutic drugs may synergistically increase therapeutic efficacy and thus represent a promising direction for research into anti-cancer therapies [178]. In addition, the combination of miRNA therapy and radiotherapy may enhance the effect of radiotherapy, inhibit the growth of cancer cells, and possibly improve the radiosensitivity of cells by altering DNA damage/repair signalling pathways [174]. Some miRNA-related cancer therapy resistance exists depending on the control of genes related to drug targets, genes related to drug pharmacokinetics, genes related to DNA damage repair, and related cell signalling pathways [179]. In addition, miRNA therapeutics can be combined with chemotherapy, radiotherapy, and immunotherapy, providing novel miRNA-based therapies, as well as the use of miRNA replacement therapy and miRNA mimetics [180]. MiRNAs, together with other ncRNAs such as circRNAs and lncRNAs, would be of great clinical importance in the pathogenesis, diagnosis, and treatment of human cancers [175,181]. In conclusion, miRNA-targeted approaches can enhance the sensitivity and efficacy of chemotherapy and radiotherapy. Their combined use could potentially help improve the prognosis of many patients with cancer [182]. Therefore, it is important to continue research on miRNAs to fully understand their mechanism of action and improve targeted cancer therapies in the future.

## Figures and Tables

**Table 1:** The signalling pathways with their identified miRNA regulators in CRC.

Signalling Pathway	miRNA	References
Wnt/β-catenin signalling pathway	miRNA-19a-3p, miRNA-34, miRNA-92a, miRNA-103/107, miRNA-135a/b, miRNA-144-3p, miRNA-148a, miRNA-150-5p/miRNA-520h, miRNA-181a-5p, miRNA-199b-3p, miRNA-200a, miRNA-203a-3p, miRNA-214, miRNA-224, miRNA-320a, miRNA-346-5p, miRNA-377-3p, miRNA-452, miRNA-490-3p, miRNA-494, miRNA-501-3p, miRNA-552, miRNA-582, miRNA-663b, miRNA-942	[94,95,96,97,98,99,100,101,102,103,104]
PI3K/AKT signalling pathway	miRNA-7, miRNA-19a, miRNA-181a, miRNA-182/-135b, miRNA-212, miRNA-193a-5p, miRNA-455-5p, miRNA-543, miRNA-761	[105,106,107,108]
STAT3 signalling pathway	miRNA-34a, miRNA-572	[109]
NF-κB signalling pathway	miRNA-410-3p	[110]
NOTCH signalling pathway	miRNA-139-5p, miRNA-195-5p	[111]
TGF signalling pathway	miRNA-18, miRNA-27a, miRNA-34a, miRNA-140-5p, miRNA-581, miRNA-1269a, miRNA-4775	[112]

**Table 2:** MiRNAs in patient serum and tumour tissue potential associated with the diagnosis or progression of colorectal cancer.

Significance	Plasma/Serum	Tissues and Cell Lines	Colon Irrigation Liquid
**present for CRC diagnosis**	miRNA-15b-5p, miRNA-17-5p, miRNA-18a-5p, miRNA-18b-5p, miRNA-19a-3p, miRNA-21 *, miRNA-23a, miRNA-25, miRNA-27a, miR-29c, miRNA-30e-3p, miRNA-92a-3p, miRNA-103a-3p, miRNA-127-3p, miRNA-146a-5p, miRNA-148a-3p, miRNA-149, miRNA-151a-5p, miRNA-181a-5p, miR-203-3p, miRNA-211, miRNA-221-3p, miRNA-223, miRNA-375, miRNA-375, miRNA-425-3p, miRNA-449a, miRNA-486-3p, miRNA-486-5p, miRNA-584-5p, miRNA-618, miRNA-762, miRNA-944, miRNA-1180-3p, miRNA-001311, miRNA-004153, miRNA-017723, miRNA-017724, miRNA-020365 [4,47,50,74,102,121,123,151,171]		miRNA-27a-3p, miRNA-124-3, miRNA-130b-3p, miRNA-421, [4,129,143,150]
**present for prognosis in CRC**	miRNA-823, miRNA-54265 [4,50]	miRNA-16 **, miRNA-20a, miRNA-21, miRNA-22, miRNA-23a-3p **, miRNA-25-3p **, miRNA-27a-3p **, miRNA-30b-5p **, miRNA-30c-5p **, miRNA-30d-5p **, miRNA-31-3p, miRNA-31-5p **, miRNA-124-1 **, miRNA-125b, miRNA-140, miRNA-143-5p **, miRNA-146a-5p **, miRNA-148a-3p **, miRNA-150-5p **, miRNA-181a-5p **, miRNA-196a-5p **, miRNA-210-3p **, miRNA-214, miRNA-215, miRNA-222-3p **, miRNA-223-3p **, miRNA-224 **, miRNA-335, miRNA-515-5p, miRNA-823, miRNA-1226-5p, miRNA1245, miRNA-24000 [4,47,60,73,81,93,99,104,120,121,123,131,147,148,160,174,175]	
**present in various types of cancers, also in CRC**		miR-27a-3p, miRNA-27b-3p, miRNA-145 *, miRNA-195, miRNA-1271 [4,60,81,93,121,130,132,133,134,137,165]	
**very low present or absent in various types of cancers, also in CRC**	miRNA-23210, miRNA-32159 [4,171]		
**very low present or absent in CRC**	miRNA-28876, miRNA-5937 [4,171]	miRNA-9, miRNA-10a, miRNA-16, miRNA-18a, miRNA-29a/b, miRNA-34a/b/c, miRNA-129, miRNA-137, miRNA-342, miRNA-345, miRNA-519, miRNA-4802 [4,57,59,73,121,142,144,145,147,170,173]	

* in clinical trials in vivo. ** poor prognosis. upregulated. downregulated.
